# Supplementary material for: A mechanistic, stigmergy model of territory formation in solitary animals: Territorial behavior can dampen disease prevalence but increase persistence
Source: PLoS Comput Biol. 2020 Jun 11;16(6):e1007457. doi: 10.1371/journal.pcbi.1007457 (PMC7289346; doi:10.1371/journal.pcbi.1007457)
Supplement: S1 Table — (DOCX) [file pcbi.1007457.s001.docx]

**S1 Table. Error rate and model accuracy from random forest models for three measured outcomes of disease dynamics.**

| **Outcome** | **Error rate (% error unexplained) for training data set** | **Model accuracy on test data set (%)** |
| --- | --- | --- |
| Outbreak success (categorical) | 28.8 | 70.8 |
| Maximum prevalence given outbreak success (regression) | 16.7 | 83.1 |
| Outbreak duration given outbreak success (regression) | 29.4 | 70.0 |
