# Supplementary figures and images for: A mechanistic, stigmergy model of territory formation in solitary animals: Territorial behavior can dampen disease prevalence but increase persistence
Source: PLoS Comput Biol. 2020 Jun 11;16(6):e1007457. doi: 10.1371/journal.pcbi.1007457 (PMC7289346; doi:10.1371/journal.pcbi.1007457)

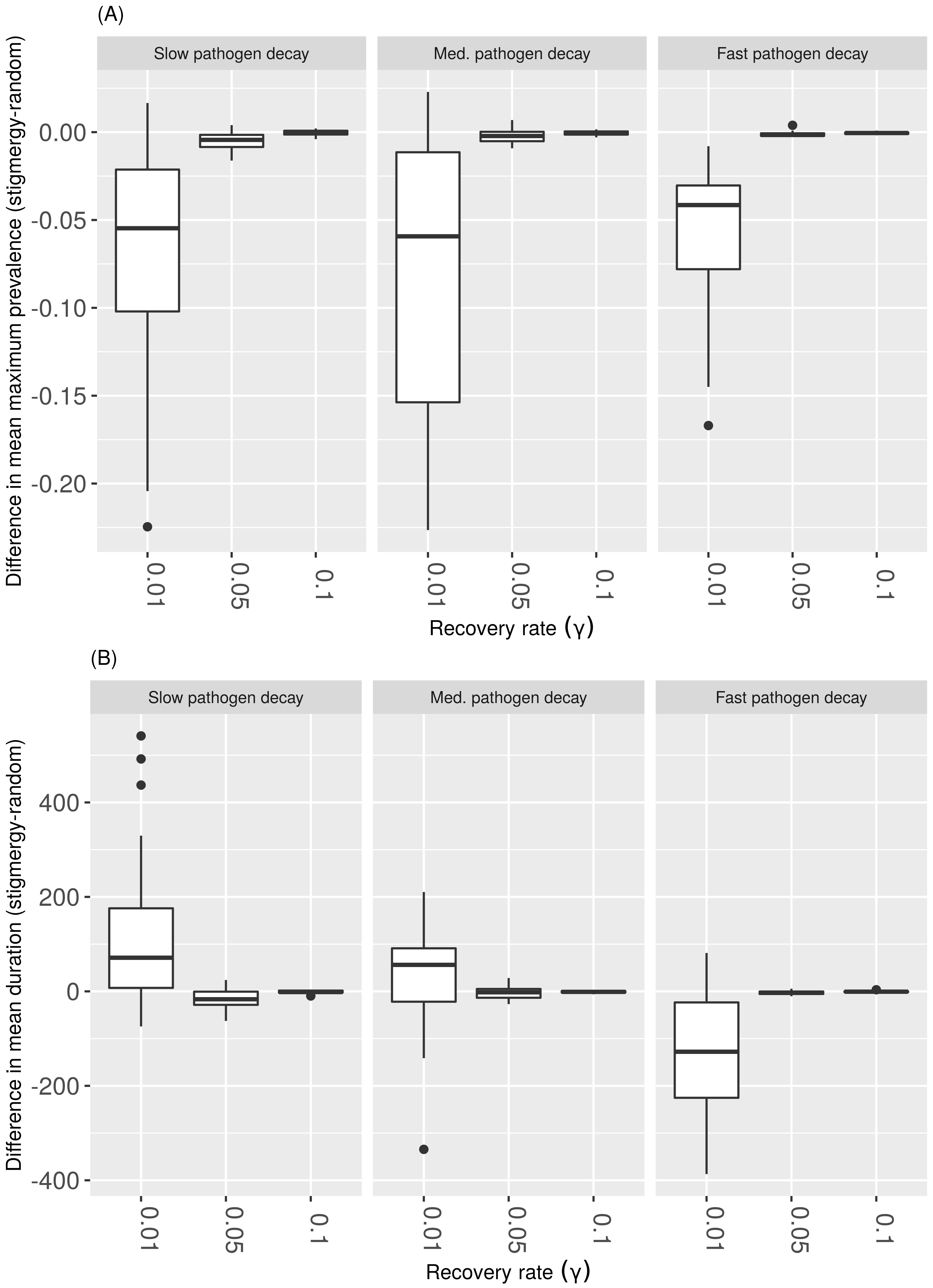

Supplement: S1 Fig — The absolute difference between stigmergy vs. random movement simulations for (A) mean maximum prevalence and (B) mean outbreak duration as a function of recovery rate and environmental decay rate of pathogen (α, columns). Each point in the box plot distribution represents a paired difference between the mean outcomes for stigmergy vs. random simulations for a given parameter set. Shown for a medium host density of 0.04 hosts/unit2. (TIF) [file pcbi.1007457.s002.tif]

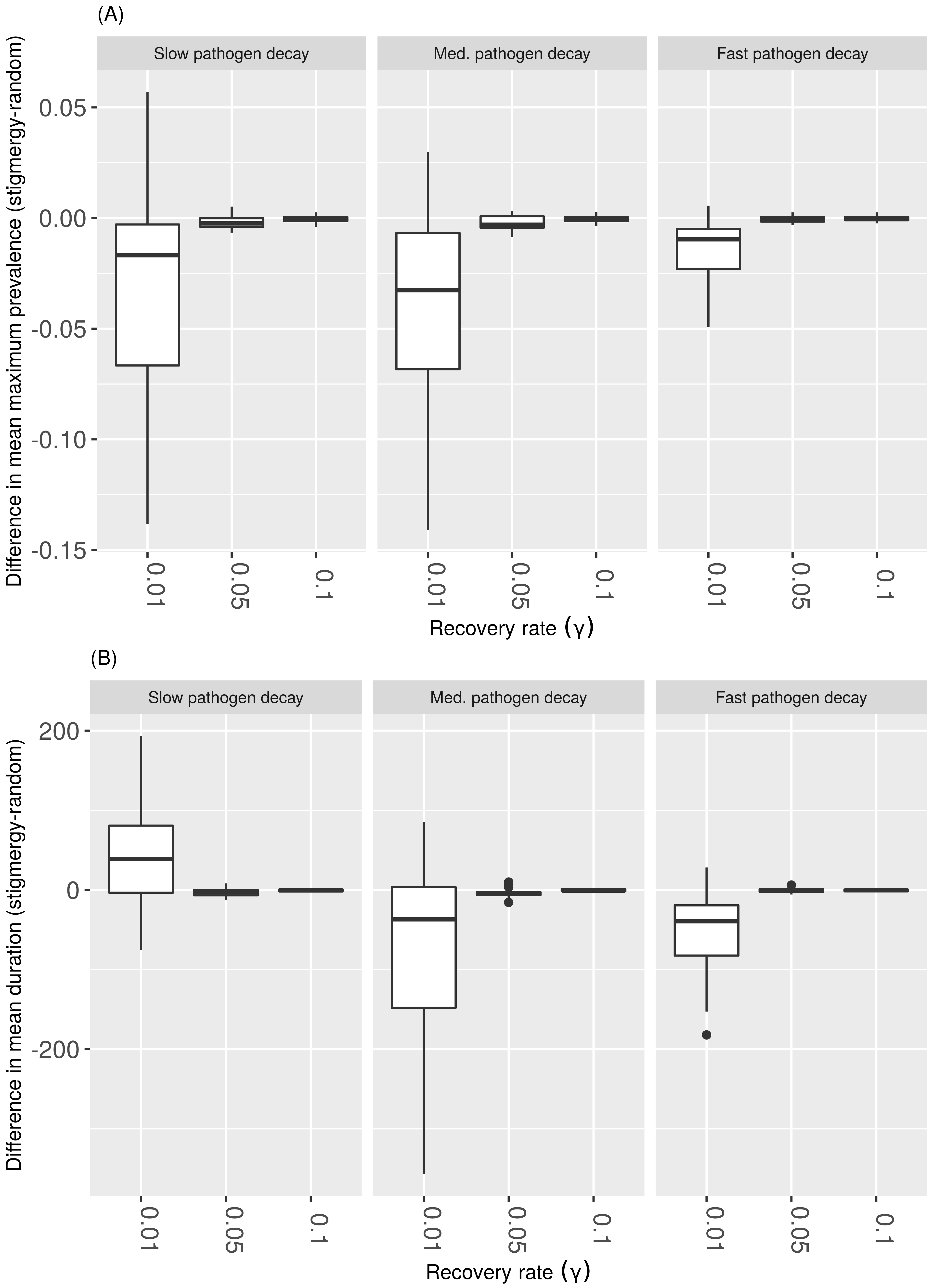

Supplement: S2 Fig — The absolute difference between stigmergy vs. random movement simulations for (A) mean maximum prevalence and (B) mean outbreak duration as a function of recovery rate and environmental decay rate of pathogen (α, columns). Each point in the box plot distribution represents a paired difference between the mean outcomes for stigmergy vs. random simulations for a given parameter set. Shown for a low host density of 0.02 hosts/unit2. (TIF) [file pcbi.1007457.s003.tif]

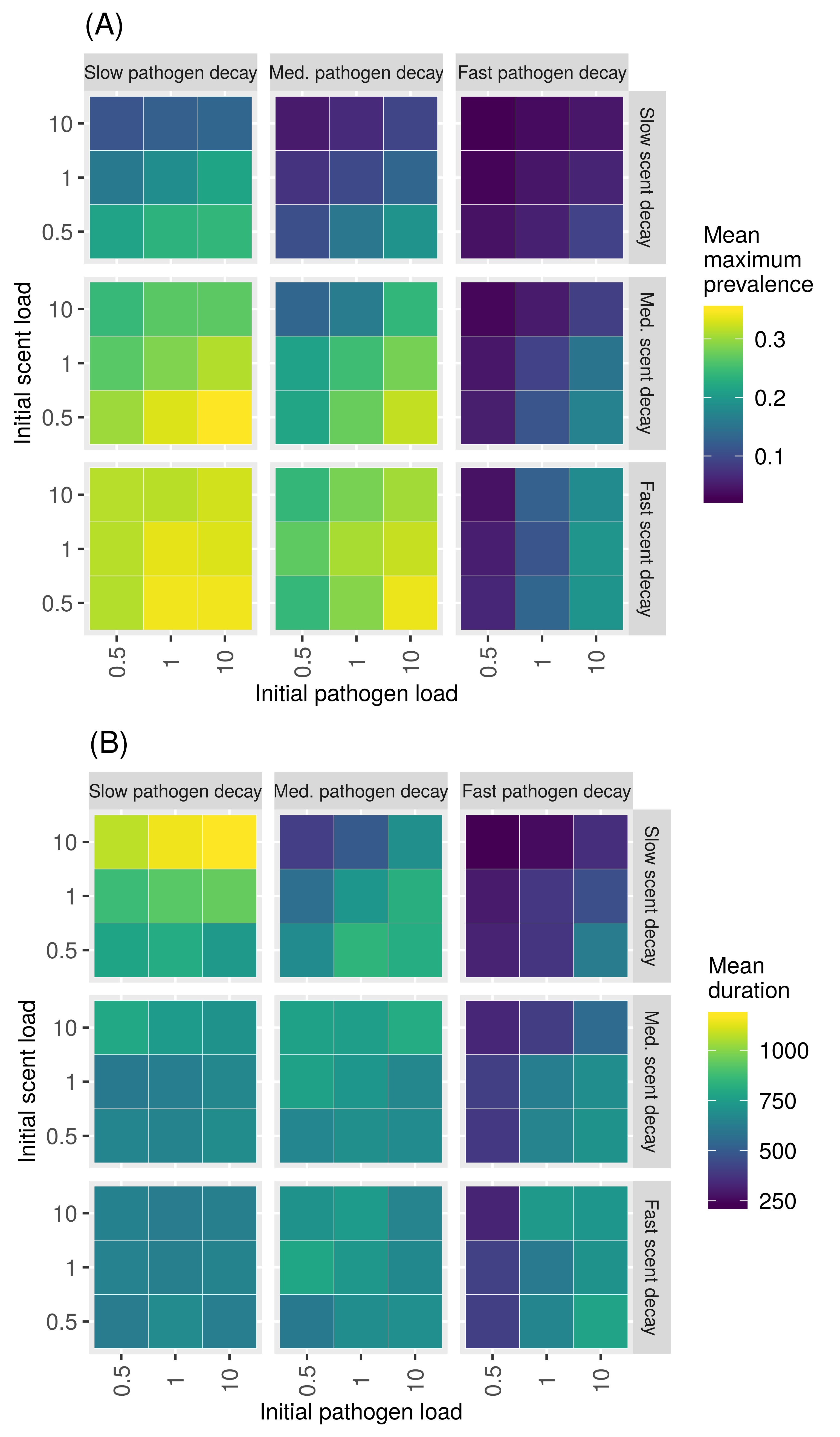

Supplement: S3 Fig — Mean maximum prevalence (A) and mean duration (B) of simulated outbreaks for simulations with a medium host density (0.04 hosts/unit2) responding to stigmergy cues with a recovery rate of 0.01/unit time. (TIF) [file pcbi.1007457.s004.tif]

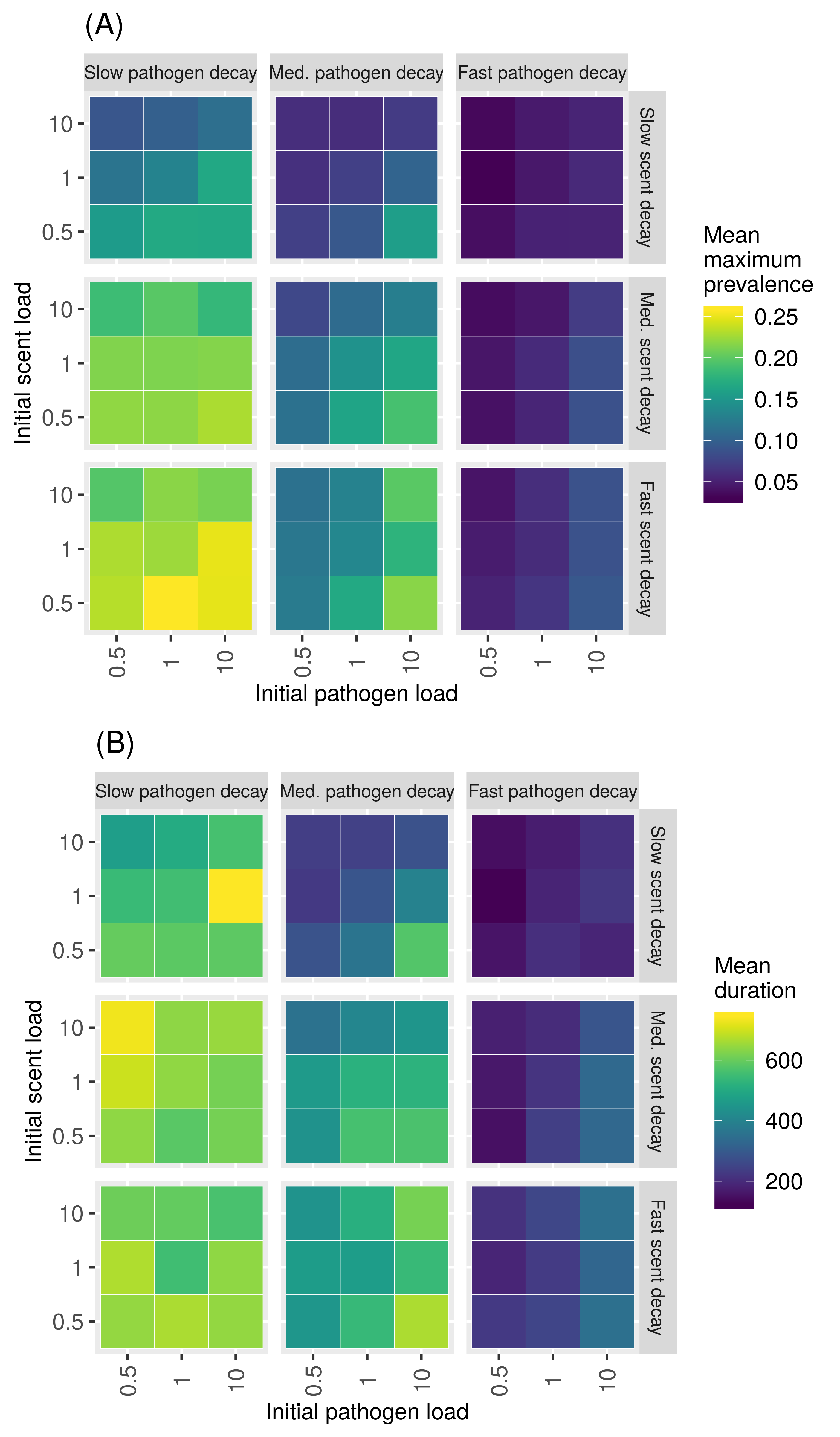

Supplement: S4 Fig — Mean maximum prevalence (A) and mean duration (B) of simulated outbreaks for simulations with a low host density (0.02 hosts/unit2) responding to stigmergy cues with a recovery rate of 0.01/unit time. (TIF) [file pcbi.1007457.s005.tif]

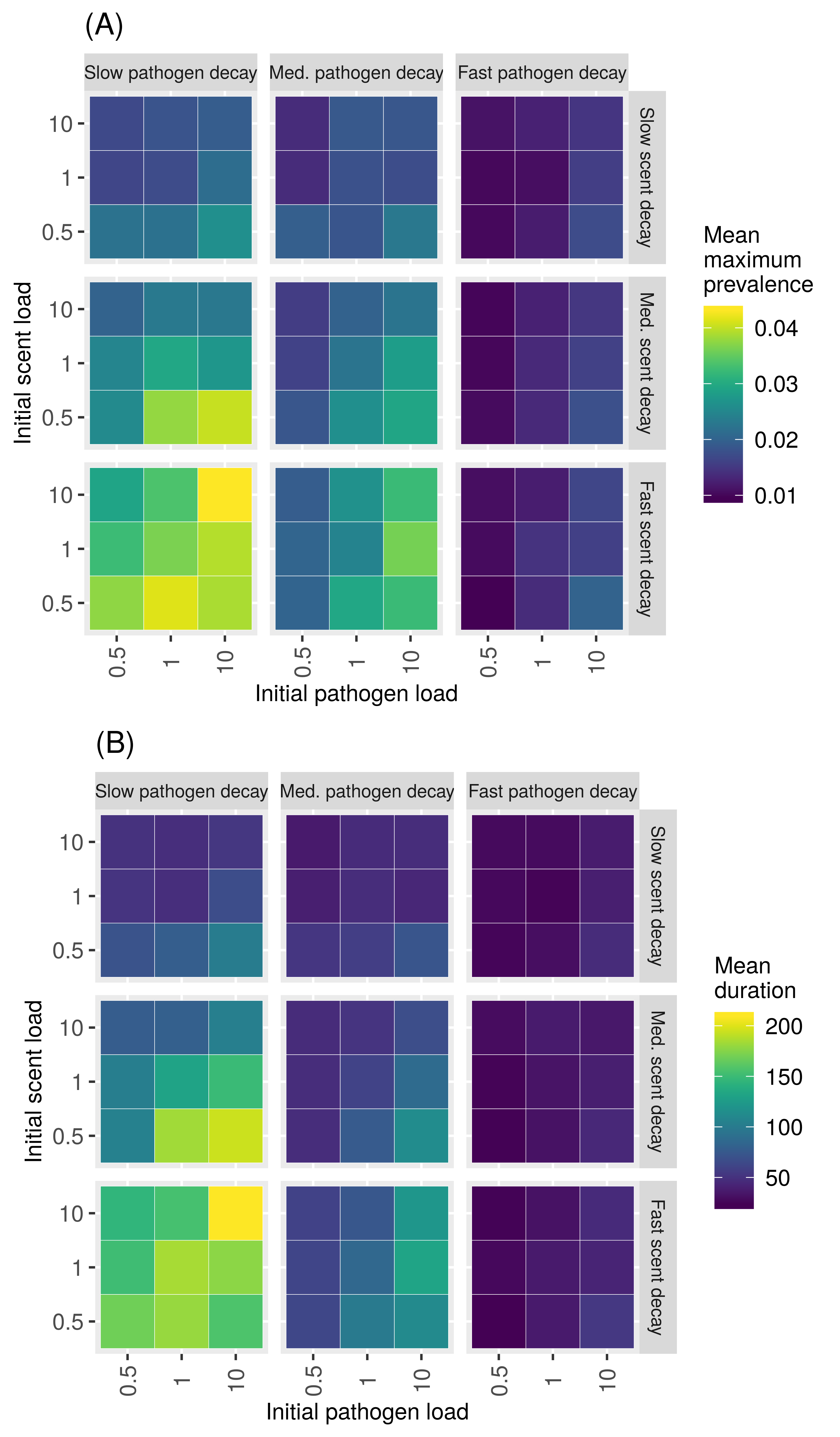

Supplement: S5 Fig — Mean maximum prevalence (A) and mean duration (B) of simulated outbreaks for simulations with a high host density (0.06 hosts/unit2) responding to stigmergy cues with a recovery rate of 0.05/unit time. (TIF) [file pcbi.1007457.s006.tif]

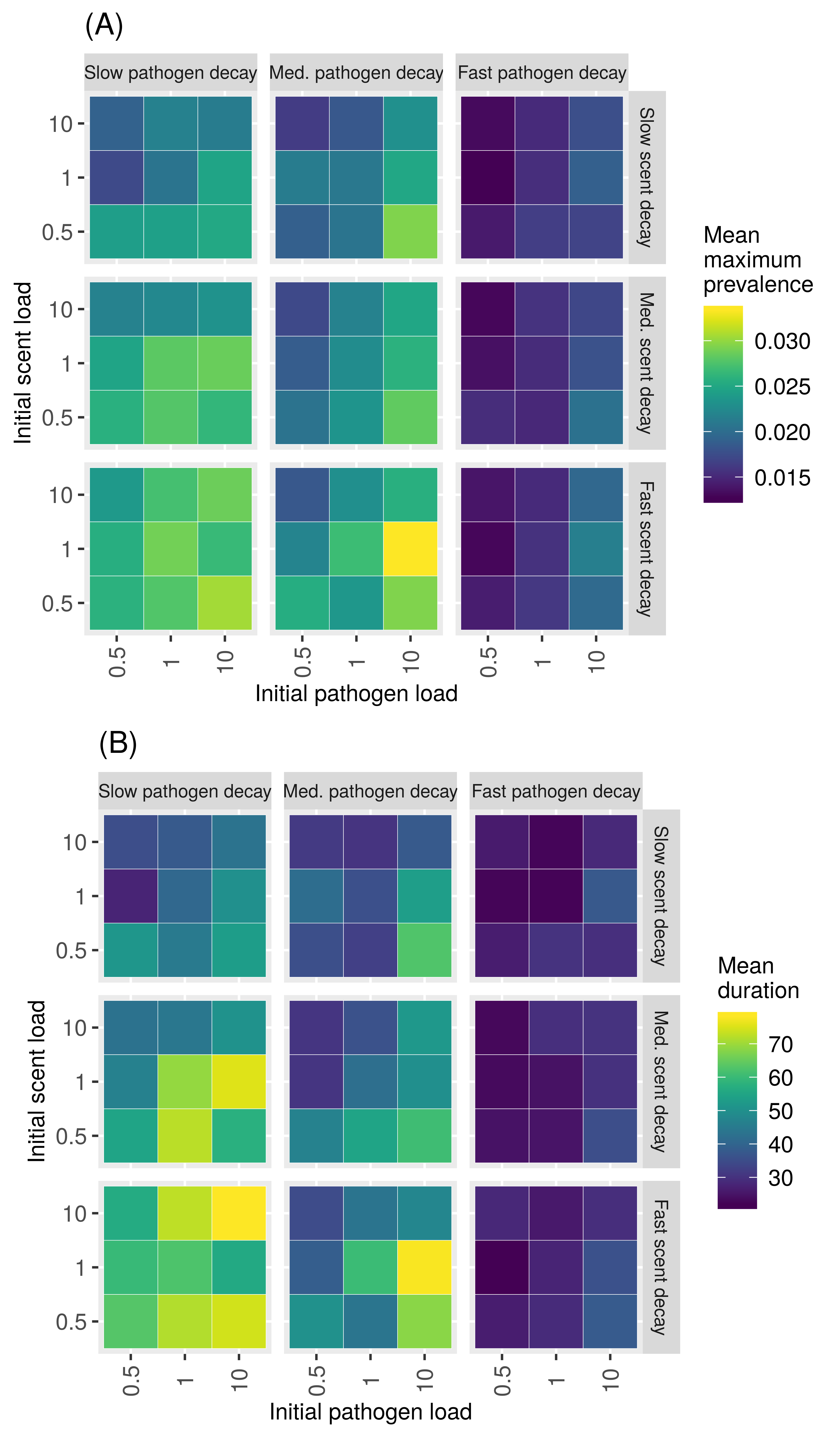

Supplement: S6 Fig — Mean maximum prevalence (A) and mean duration (B) of simulated outbreaks for simulations with a medium host density (0.04 hosts/unit2) responding to stigmergy cues with a recovery rate of 0.05/unit time. (TIF) [file pcbi.1007457.s007.tif]

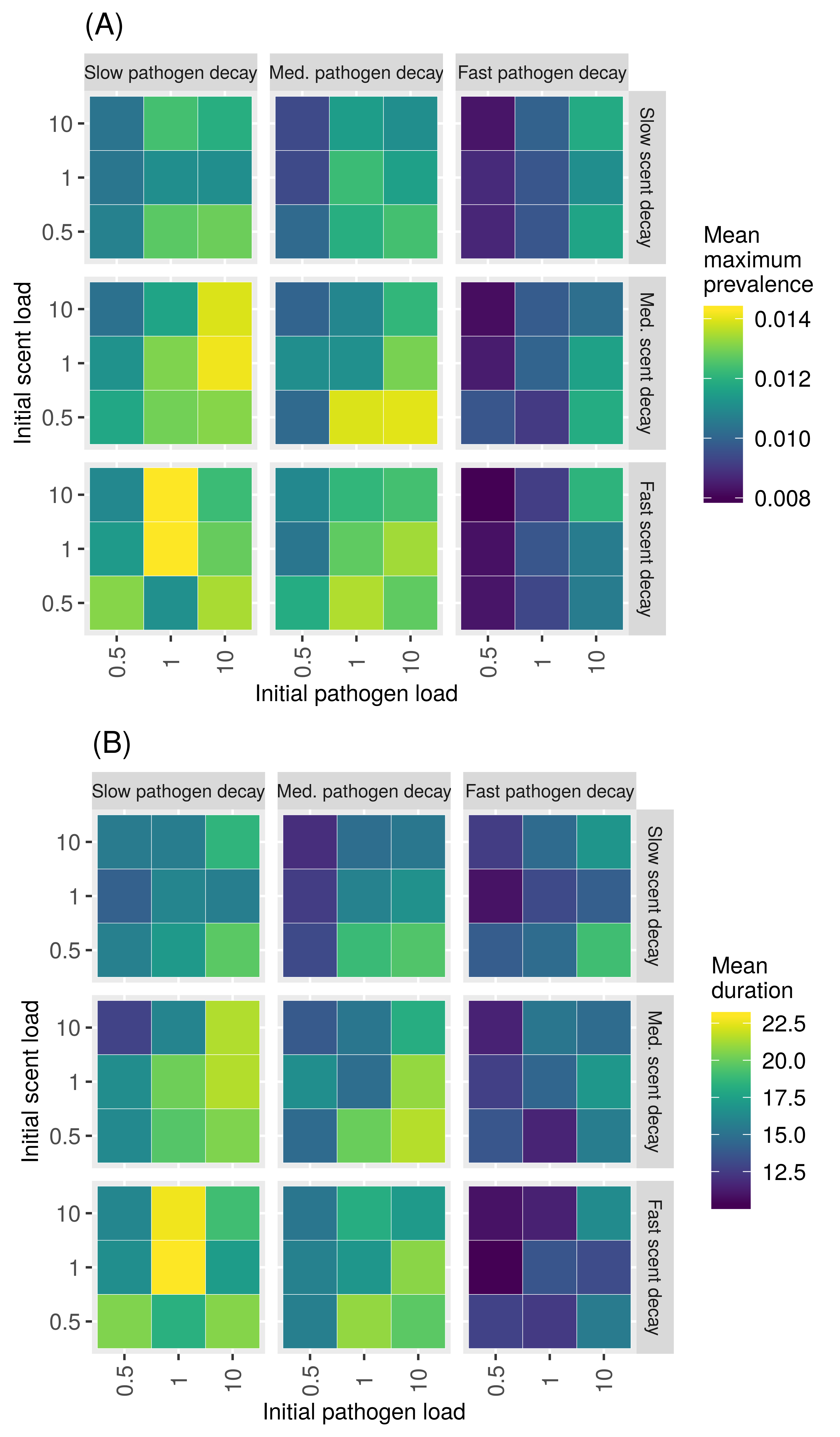

Supplement: S7 Fig — Mean maximum prevalence (A) and mean duration (B) of simulated outbreaks for simulations with a high host density (0.06 hosts/unit2) responding to stigmergy cues with a recovery rate of 0.10/unit time. (TIF) [file pcbi.1007457.s008.tif]

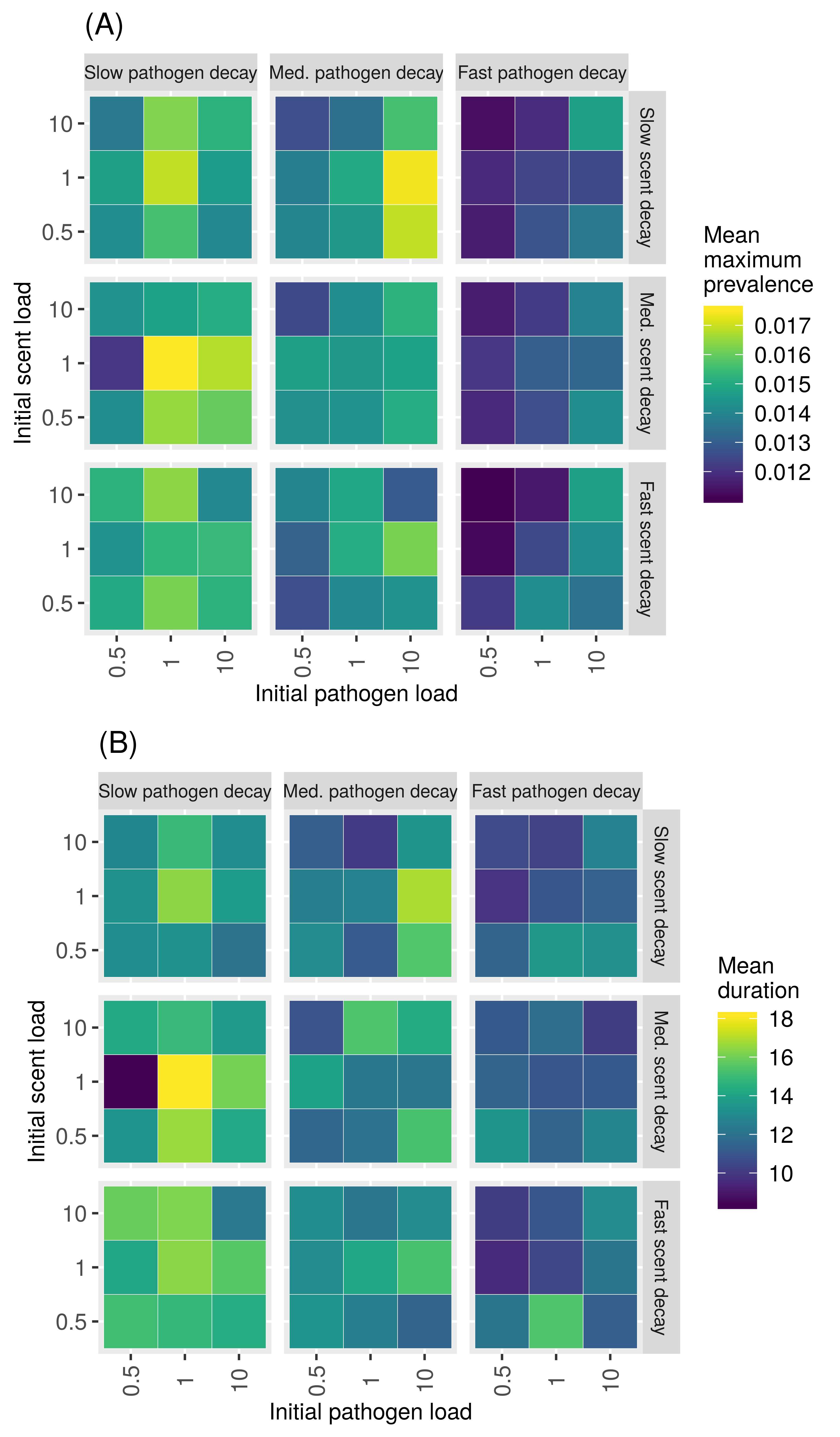

Supplement: S8 Fig — Mean maximum prevalence (A) and mean duration (B) of simulated outbreaks for simulations with a medium host density (0.04 hosts/unit2) responding to stigmergy cues with a recovery rate of 0.10/unit time. (TIF) [file pcbi.1007457.s009.tif]

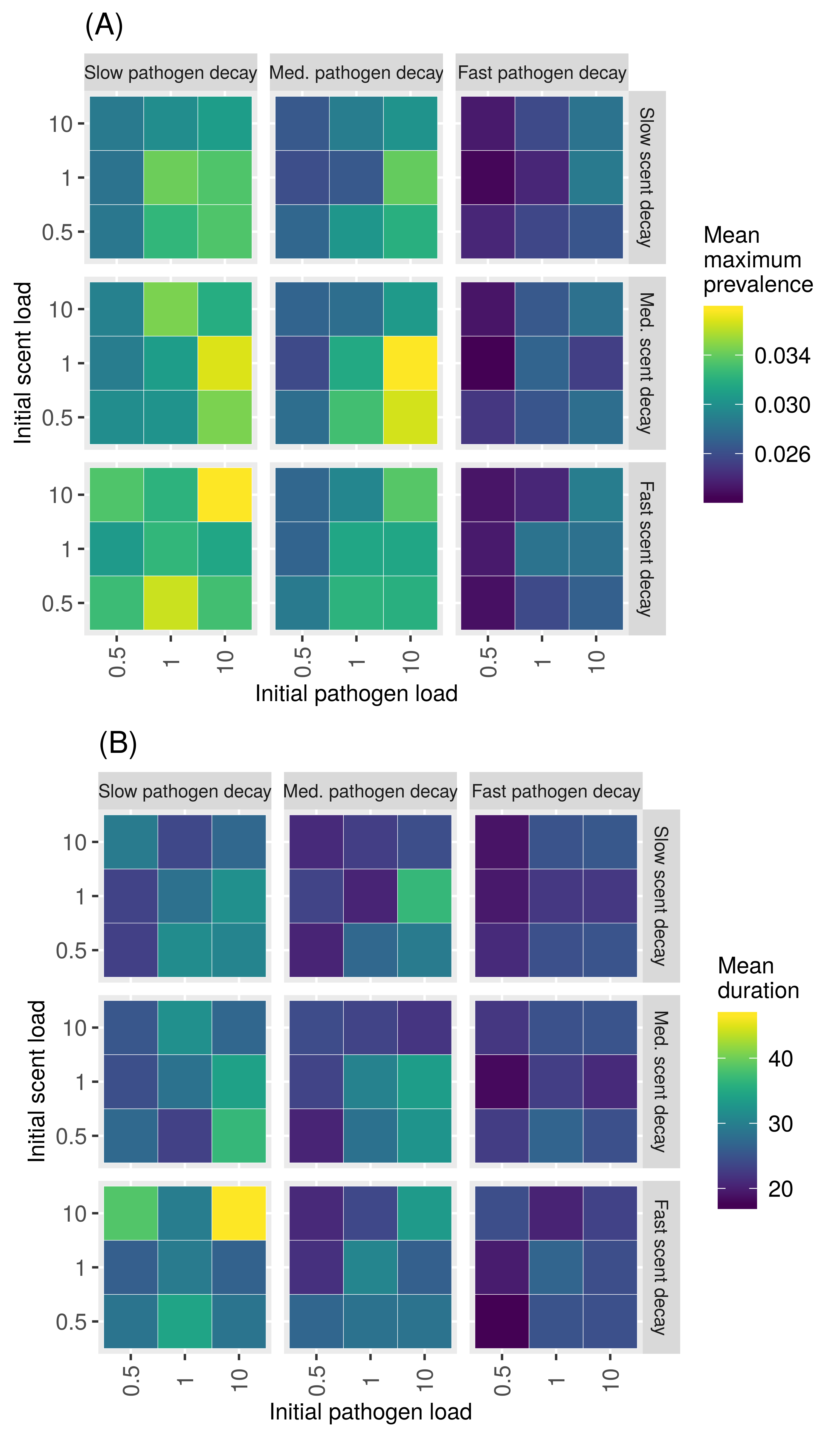

Supplement: S9 Fig — Mean maximum prevalence (A) and mean duration (B) of simulated outbreaks for simulations with a low host density (0.02 hosts/unit2) responding to stigmergy cues with a recovery rate of 0.05/unit time. (TIF) [file pcbi.1007457.s010.tif]

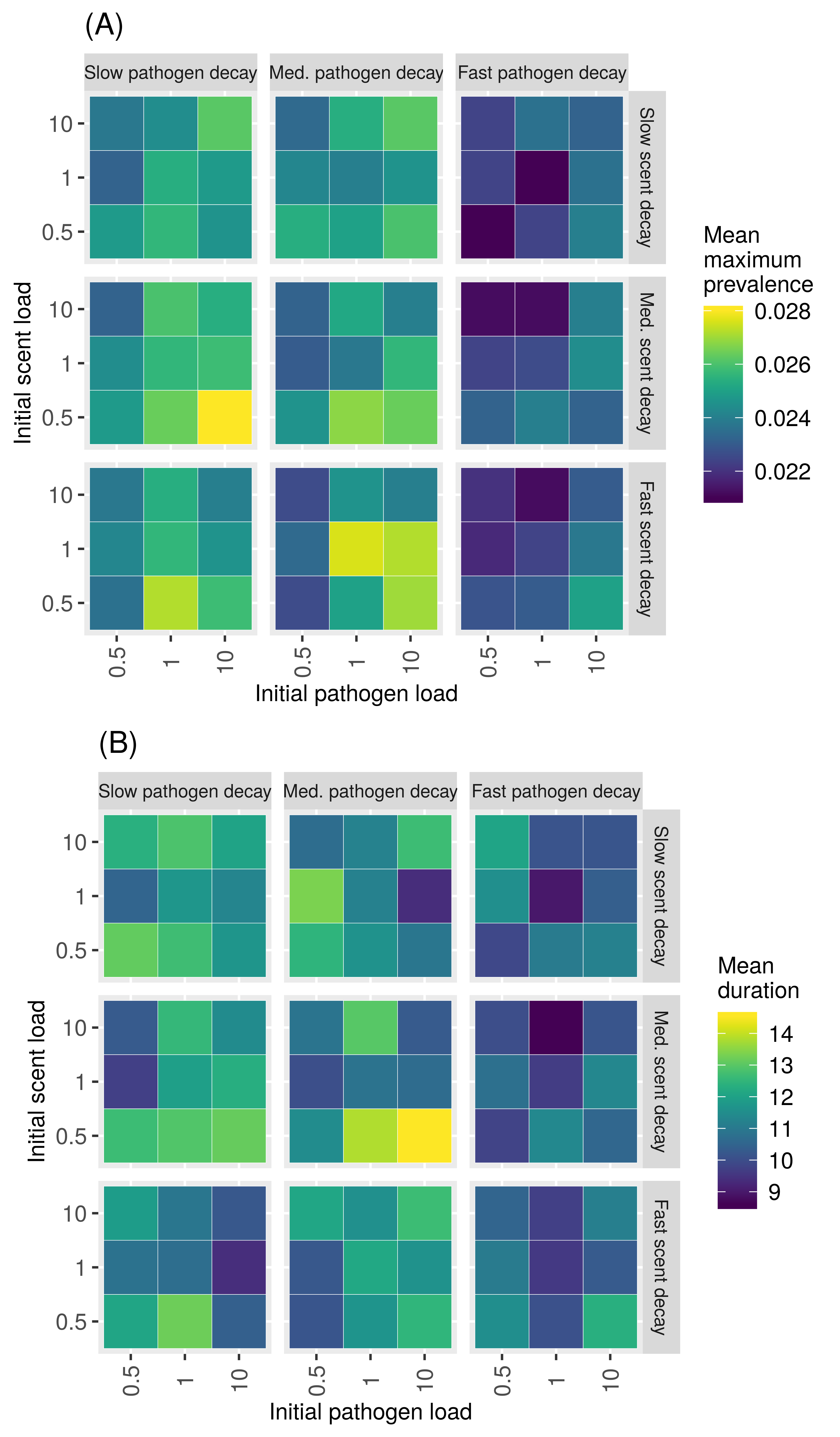

Supplement: S10 Fig — Mean maximum prevalence (A) and mean duration (B) of simulated outbreaks for simulations with a low host density (0.02 hosts/unit2) responding to stigmergy cues with a recovery rate of 0.10/unit time. (TIF) [file pcbi.1007457.s011.tif]

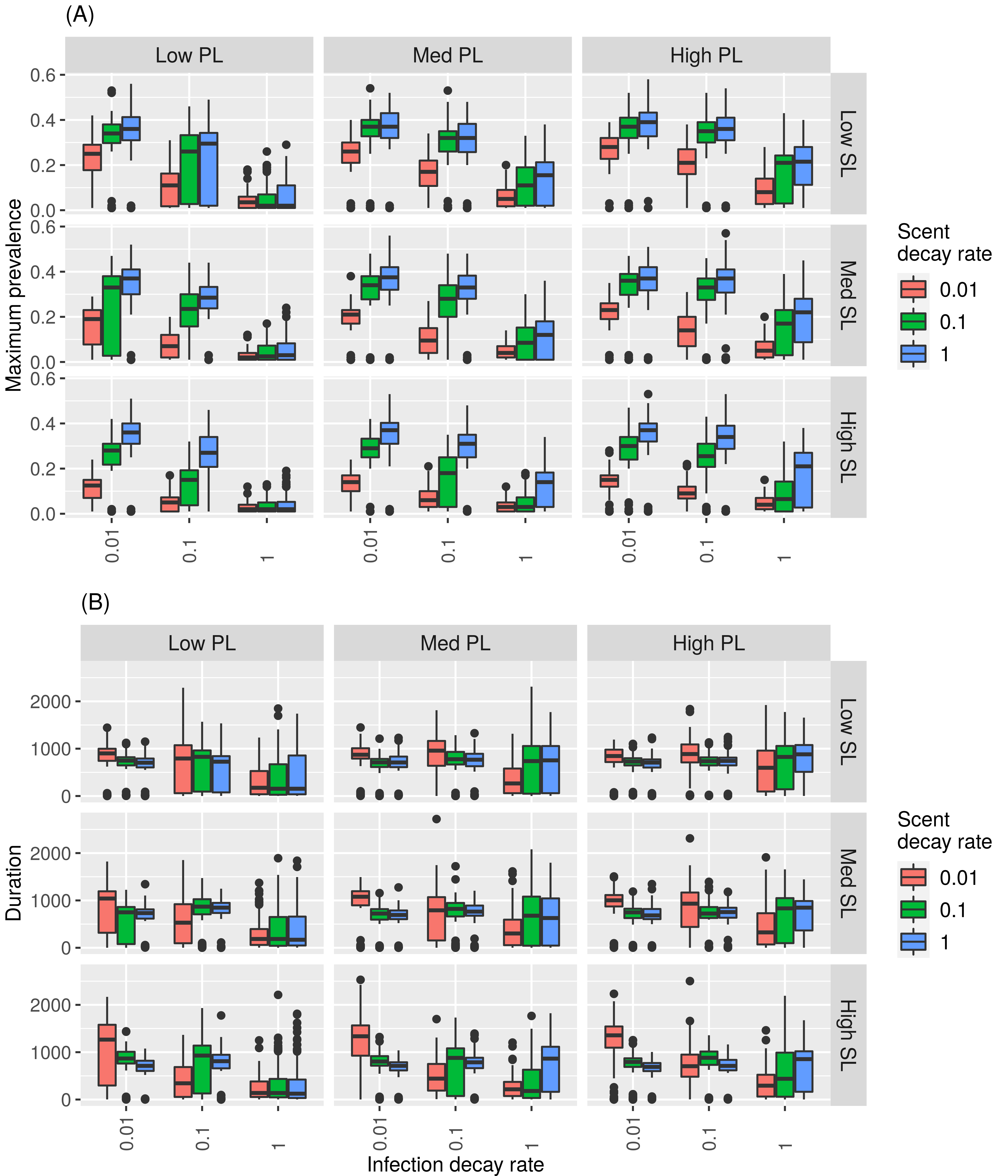

Supplement: S11 Fig — Boxplots of (A) maximum prevalence and (B) outbreak duration with a medium host density (0.04 hosts/unit2) responding to stigmergy cues and a recovery rate of 0.01/time step. Rows correspond to low, medium, and fast scent loads (SL). Columns correspond to low, medium, and fast pathogen loads (PL). (TIF) [file pcbi.1007457.s012.tif]

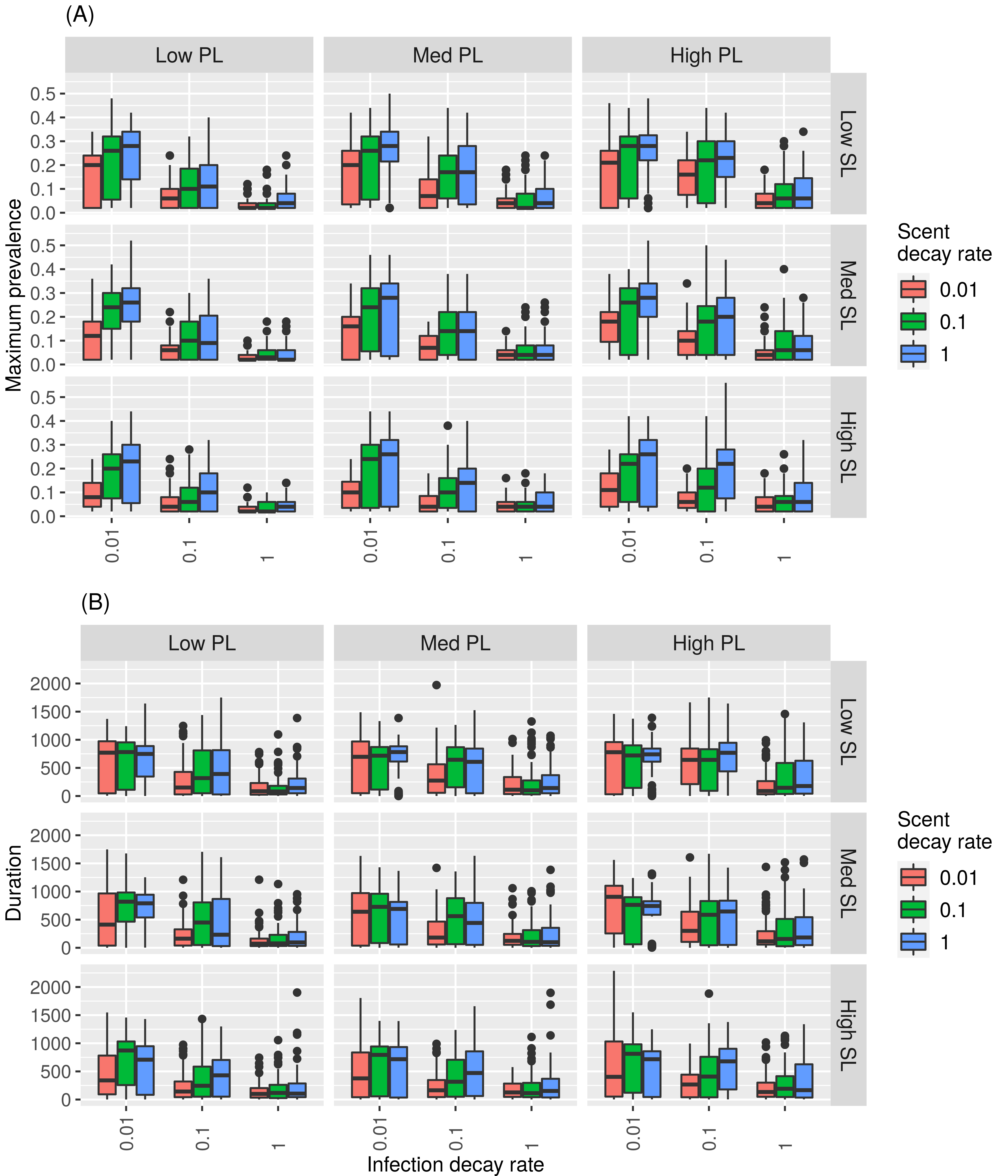

Supplement: S12 Fig — Boxplots of (A) maximum prevalence and (B) outbreak duration with a low host density (0.02 hosts/unit2) responding to stigmergy cues and a recovery rate of 0.01/time step. Rows correspond to low, medium, and fast scent loads (SL). Columns correspond to low, medium, and fast pathogen loads (PL). (TIF) [file pcbi.1007457.s013.tif]
