# Supplementary material for: A mechanistic, stigmergy model of territory formation in solitary animals: Territorial behavior can dampen disease prevalence but increase persistence
Source: PLoS Comput Biol. 2020 Jun 11;16(6):e1007457. doi: 10.1371/journal.pcbi.1007457 (PMC7289346; doi:10.1371/journal.pcbi.1007457)
Supplement: Attachment — Submitted filename: Stigmergy Response To Reviewers.docx [file pcbi.1007457.s014.docx]

January 23, 2020

Dear Dr. Ferrari and Dr. Pitzer,

Thank you for the opportunity to submit a revised version of our manuscript entitled, “A mechanistic, stigmergy model of territory formation in solitary animals: Territorial behavior can dampen disease prevalence but increase persistence” (PCOMPBIOL-D-19-01705) to *PLOS Computational Biology*. We thank you and the reviewers for taking the time to review the manuscript, engage with it, and provide constructive feedback. We have accordingly made appropriate changes to the manuscript to address these comments, which we think have substantially improved our work. We present responses to individual points below.

For clarity and ease of reference, we have numbered reviewer comments sequentially below and we highlighted changes in yellow in the manuscript.

With these modifications, we think our manuscript would be a valuable and timely addition for the cross-journal collection on Mathematical Modelling of Infectious Disease Dynamics.

Sincere thanks for your consideration,

Lauren A. White, PhD

I thank the authors for their manuscript. Both reviewers have made helpful suggestions for changes that I believe will improve the clarity of the presentation. In particular:

R1 has asked for some clarification in the presentation of absolute differences in model outputs as a function of random vs. stigmergy movement. Further, R1 has asked for more evidence in support of the argument that "territoriality “may increase the likelihood of pathogen persistence”. As I agree with the reviewer that this is an interesting conjecture, I would encourage the authors to make every attempt to strengthen the support for this claim using the evidence presented.

***Response: We have adopted Reviewer 1’s strategy for visually demonstrating the absolute differences in random vs. stigmergy movement (notably Figures 4, S1 & S2). This approach has substantially strengthened our claim that territoriality “may increase the likelihood of pathogen persistence.”**

R2 has offered a number of suggestions for links to the existing literature that the authors should carefully consider. Further, I agree with R2 that the inclusion of the material in L141-204 in "results" is awkward. While we're at the mercy of the PLoS template, I have handled many papers that include a model description section between the introduction and results; I would recommend such a reorganization here. Further, I encourage the authors to clarify the transmission model per the request of R2.

***Response: In response to the comments from Reviewer #2, we have better contextualized this study within the realm of existing literature (particularly see responses 2.1, 2.3, and 2.8 for more detail). We have also updated the organization of the manuscript to include a “Model description” section per your suggestion (starting on line 128). Finally, we have further clarified the transmission model (lines 195-218).**

**Reviewer's Responses to Questions**

**Reviewer #1:** In this manuscript, White et al. develop an individual based model that describes the dynamics of an environmentally transmitting pathogen through a population of asocial species where individuals display territorial movement. The main conclusion of the manuscript is that the duration of infectious period, population density and pathogen decay rate are the most important factors that influence outbreak severity, which is to be expected. Territoriality only influences outbreak severity when the conditions are conducive to outbreaks. For these scenarios, the authors report that territoriality reduces peak prevalence and may “increase the likelihood of pathogen persistence”. The model developed in this paper is interesting and important. It can be possibly be expanded in future to explore the impact of social behavior by modeling movement that is in between random and asocial territorial (but, of course, this is beyond the scope of the current manuscript). I have a few concerns that I would like to see better caveated in the manuscript or addressed with changes to the analysis if feasible.

1.1) Analysis of the impact of territoriality on outbreak severity: Much of the results about the effects of territoriality relies on qualitative difference between pooled results for random and stigmergy movement (for example, difference between top and bottom panel of Figure 4). I wonder if these results can be strengthened by instead reporting delta maximum prevalence and delta outbreak duration (where delta maximum prevalence = maximum prevalence (stigmergy movement) - maximum prevalence (random movement) for the same parameter combination). This approach would allow direct one-to-one comparison of how territorial movement would influence outbreak dynamics under similar parameter combination.

***Response: Thank you for your helpful suggestions about how to better present the observed differences in outbreak outcomes for stigmergy vs. random movement. We have updated Figures 4, S1 & S2 as box plots where the distribution is the absolute difference in mean maximum prevalence or mean duration for a given parameter set. Not only does this simplify the number of panels per figure (per comment 2.2b below), it also highlights key findings, namely, that stigmergy driven movement can decrease outbreak size and increase outbreak persistence within certain parameter spaces.**

1.2) I also worry about the statement (in abstract and rest of the manuscript) that territoriality “may increase the likelihood of pathogen persistence” follows the finding that territoriality, under certain conditions makes the “outbreak duration considerably more variable”. Increase in variation around the mean does not imply that likelihood of pathogen persistence is higher. In fact, increase in variation implies that it is equally likely that the likelihood of pathogen persistence may be reduced. I believe that my previous suggestion of reporting values for delta outbreak duration will help to evaluate whether territoriality tends to increase or decrease outbreak duration.

***Response: Thank you for these suggestions that help clarify our message. After taking the suggested approach from comment 1.1 above, we are confident that simulations with high population density, slower recovery rate, and slower pathogen decay rate are, in fact, longer lasting than corresponding simulations with random movement (per Figure 4). We have updated our language in the abstract (lines 50-51), results (line 276), and discussion (lines 324-425) to more specifically reference these patterns and removed the non-sequitur of outbreak duration variability.**

1.3) Variable importance analysis: was the model performance evaluated using test data set? What was the prediction error of the model? Please show these results.

***Response: We now detail the methodology of using test data sets (lines 434-441) and report prediction error for training sets and model accuracy for test data sets (lines 607-609, S1 Table).**

1.4) Line 229 – 236: Effect of stigmergy on outbreak duration: The authors report that territorial movement increases disease persistence in certain cases. However, looking at Figure 4b, it seems that there are only a handful of outlier cases were outbreak duration due to stigmergy is higher compared to random movement (please also see my comment on this issue above).

***Response: We have revised our presentation of results to strengthen our conclusions. By looking at absolute differences for paired stigmergy vs. random movement simulation parameter sets (per comment 1.1 above), we have improved our data presentation to clearly illustrate that territorial movement can increase outbreak length in some cases relative to random movement — particularly for slower pathogen decay rates (Fig 4, S1 & S2 Figs).**

Minor comments:

1.5) This is a minor point, but using “population size” and “population density” for the same parameter is confusing. I suggest that the authors stick to using one terminology throughout the manuscript.

***Response: Thank you for this excellent comment. We have updated the text to refer to host density throughout the text (e.g., lines 285, 287, figure legends).**

1.6) Discussion, line 289: I believe it should be “lower initial scent load … promoted higher maximum prevalences” instead of “higher initial scent load”

***Response: Thank you for this observation. We have modified the text to read, “lower initial scent loads paired with faster scent decay promoted higher maximum prevalences” (now line 331).**

1.7) Line 212: (Fig 3B&C) instead of (Fig 3A&B).

***Response: Thank you for this observation. We have corrected this mislabeling (now line 253).**

**Reviewer #2:** This paper addresses the important topic of movement-driven disease transmission in a population of territorial animals. It combines a general individual-based model of conspecific-avoidant territorial movement with an SIR model. While its findings were not surprising, the model is valuable for its explicit integration of disease and behavioral dynamics. However, a lot of mechanistic details are left unexplained in the manuscript, particularly the assumptions made about the transmission process. In addition, there is a notable gap in the citations of relevant literature; critically, it overlooks several recent key theoretical works that are integral to the scope of the present study. Lastly, the manuscript needs to be substantially reorganized – currently, majority of the method description, which is also oddly attached at the end, is relegated to Results. All these problems could be addressed in the revision.

***Response: Thanks for your constructive feedback. To address these concerns, we have:**

- **Provided additional detail about the formulation of the model, particularly the transmission process (lines 195-219) and its relation to previous work (lines 183-193).**
- **Updated the literature in the Introduction to better reflect the current state of the field (in particular, see our responses to comments 2.3 and 2.8 below).**
- **Moved the methods to a “Model description” section in front of the results (beginning on line 128). Per the editor’s suggestion, this reordering should make the flow of the text more linear, but still consistent with PLOS formatting standards.**

Major problems:

2.1) The model seems to be heavily influenced by recent mechanistic territory models by Potts, Giuggioli et al. However, it has significantly simplified their descriptions of the movement process, e.g. assuming no directional bias, centralizing tendency, or spatial autocorrelation. The formulation of the movement kernel also deviates from many past models, which often assume distinct distributions of step length and directionality (see Moorcroft and Lewis 2006). Clarifying these differences in the method section would help to put this work in context. More importantly, the entire mathematical description of the SIR model is missing from the main text. It includes essential information that would drastically alter the predictions, e.g. how density-dependent transmission is modeled. In other words, more equations need to be shown.

***Response: Thanks for pointing out some omissions in our description of this model. We have further explained the model in the context of prior work in the new “Model description” section (lines 183-193). We have also updated our description of the indirect disease transmission process (lines 195-219) per** [**Lanzas et al. (2020)**](https://paperpile.com/c/Nmqa61/U20j)**.**

2.2a) The paper's organization is extremely confusing. In the Results section, everything from lines 141-204 belongs in Methods, which should also include lines 372-406.

***Response: Per the editor’s suggestion, we have restructured the paper to include a “Model description” section prior to the results (beginning on line 128). We hope this resolves any confusion surrounding organization and flow of the manuscript.**

2.2b) Each major figure (Figs 4-6) has 12-18(!) panels, which is way too complex. I strongly recommend developing alternative visualization that compresses the most essential information down to at most 4 panels per figure. Moreover, the effects of population density on movement-dependent disease spread is an especially interesting problem that could be more extensively explored here.

***Response: Thank you for the figure insights. For the presentation of the results, we have simplified Figure 4 to six panels (based on comment 1.1 above). Likewise, for Figures 5 & 6 we have eliminated the “middle” scenarios of medium scent decay and pathogen decay rate or scent load and pathogen load. We hope this is a reasonable compromise of simplifying figure presentation while capturing some interactions between multiple parameters.**

**Reviewer 1’s suggestions about how to display differences between stigmergy and random movement have helped flush out our discussion surrounding the role of host density. In particular, we now highlight some interesting interactions between host density, pathogen decay rate, and outbreak duration (lines 277-280).**

Minor problems:

2.3) L38 – mechanistic models of animal territoriality, or more generally, home range formation, have been developed since the 80s (see Okubo 1980). See below for some recent works relevant to this manuscript.

***Response: We have rewritten this introductory sentence to the abstract as: “Although movement ecology has leveraged models of home range formation to explore the effects of spatial heterogeneity and social cues on movement behavior, disease ecology has yet to integrate these potential drivers and mechanisms of contact behavior into a generalizable disease modeling framework” (lines 38-41).**

2.4) L39 – collective movement is more commonly considered as a subset of movement ecology.

***Response: Thank you for this clarification. In the reworking this sentence (see comment 2.3 above), we have removed this particular clause.**

2.5) L41 – even exclusive territoriality does not equate to asociality; territorial maintenance typically requires direct or indirect conspecific interactions, which all involve social responses.

***Response: Thank you for this observation. We have replaced the word “asocial” with “solitary” in the title (line 6), abstract (lines 42), and main text (line 96, 385).**

2.6) L63-64 – this is not accurate; plenty of empirical and theoretical works suggest otherwise

***Response: We have removed this sentence from the Author Summary.**

2.7) L77 – edit: “According to the general conceptual framework of movement ecology proposed by Nathan et al”

***Response: We have updated the text to read: “According to the general conceptual framework proposed by Nathan et al. (2008), there are four motivating questions for movement ecology research: ...” (lines 78-79).**

2.8) L88 – Not accurate; notably, see Moorcroft et al. (Proc B 2006), Wang and Grimm (Ecol Modelling 2007), Bateman et al. (J Animal Ecology 2015), Tao et al. (American Naturalist, 2016), and Potts et al. (Theoretical Ecology, 2019).

***Response: Thank you for these suggestions to better represent existing literature in our introduction. We have accordingly rewritten these introductory sentences as: “In movement ecology, mechanistic models of home range formation have their roots in a spatially-biased random walk process (Okubo and Levin 2001; Bateman et al. 2015). These models have since evolved to incorporate underlying resource availability and selection, population dynamics, and territorial behaviors like scent marking that lead to dynamic home range formation (Wang and Grimm 2007; Tao et al. 2016; Potts et al. 2019; Giuggioli et al. 2011; Giuggioli et al. 2013) leading to individual interactions” (lines 88-91).**

2.9) L99 – edit: “lacks strong empirical support”

***Response: We have made the suggested change (line 100).**

2.10) L112-115 – I suggest reordering the sentences

***Response: We have switched the order of these sentences (lines 113-116).**

2.11) L116-129 – population thresholds and host-pathogen coevolution are very much outside the scope of the present study; this material should be moved to the Discussion.

***Response: We have moved this paragraph from the Introduction to the Discussion section (now lines 343-356).**

2.12) L163 – this constraint only occurs probabilistically according to L172

***Response: Thank you for pointing out this discrepancy. We have updated the Figure 1 legend to be consistent with the main text in the “Model description” section. This sentence now reads: “In the case of encountering a conspecific cue (open green circle), the host has some probability of constraining its next step to 45 degrees on either side of the prior direction of movement (indicated by the directions of the red arrows); this probability depends on the strength of the conspecific cue that the host encounters” (lines 159-162).**

2.13) L169 – don’t italicize min

***Response: “min” is no longer italicized (lines 167, 169, 173, 216).**

2.14) L172 – italicize p and all mathematical variables

***Response: All instances of *p* and other mathematical variables have been italicized (e.g., Table 1, lines 146-147, 173 in “Model Description”).**

2.15) L193 – this part of the model wasn’t discussed earlier

***Response: We have now clarified much earlier in the “Model Description” that we are simulating both stigmergy-driven and random movement (lines 131-132) and then clarify how stigmergy-driven movement differs from random movement(beginning line 148).**

2.16) L195 – so an outbreak that only affects two individuals before burning out would count as a success?

***Response: This is the correct interpretation of our assumption. This is obviously an arbitrary threshold, but is useful in cases (as in this simulation context), where there are a high proportion of unsuccessful transmission events (i.e., zero inflation).**

2.17) L197 – why only model max prevalence, not mean? Justification needed.

***Response: We have updated this sentence to read correctly that we are taking the mean outcome of 100 simulations for a given parameter set: “we recorded mean outbreak success (did the disease spread beyond the initial index case?), mean maximum prevalence, and mean outbreak duration (the number of time steps until there were no remaining infectious individuals on the landscape)” (lines 233-235).**

**We look at maximum prevalence and outbreak duration because this tells us more about the shape of the outbreak curve. If we just calculate mean prevalence over time, this does not necessarily distinguish between outbreaks that spread through the entire population and die out quickly vs. those that spread slowly but persist for longer periods.**

2.18) Fig 3 – subtitles needed (i.e. outbreak likelihood, maximum prevalence, outbreak duration); spell out the variable names on the y axis and keep the same order for all three panels for visual clarity; remove %IncMSE from x label.

***Response: We have added subtitles, spelled out parameter names on the y-axis, and removed “%IncMSE” from the x-axis label (Fig 3). We have kept the parameter order in decreasing order of variable importance because this is a primary illustrative goal of the figure.**

2.19) L225-226 – confusing wording

***Response: Based on this feedback and Reviewer 1’s suggestions about how to present results (comment 1.1 above), we have updated Figure 4 legend to read: “The absolute difference between stigmergy vs. random movement simulations for (A) mean maximum prevalence and (B) mean outbreak duration as a function of recovery rate and environmental decay rate of pathogen (**$\alpha\boldsymbol{,}$**columns). Each point in the box plot distribution represents a paired difference between the mean outcomes for stigmergy vs. random simulations for a given parameter set” (lines 263-267).**

2.20) L385 – edit: “The probability of an individual relocating from x’ to x is determined by a movement kernel …”; also, note that the area under this kernel does not equal to one. Use conventional mathematical grammar in this paragraph (use e.g. Moorcroft and Lewis 2006, Bateman et al. 2015, as templates)

***Response: We have updated our description of the movement process accordingly and noted that the area under the movement kernel does not equal one (lines 132-147).**

2.21) L389 – why a uniform distribution instead of a Gaussian or the conventionally used Von Mises distribution?

***Response: Since we were investigating many other dimensions surrounding the pathogen transmission process, we wanted to use the simplest version of a distribution. Note that, in particular, the Von Mises distribution simplifies to a uniform circular distribution (no preferred directionality) when the concentration parameter approaches zero** [**(Moorcroft & Lewis, 2006, p. 24)**](https://paperpile.com/c/Nmqa61/ylIl/?locator=24)**. We have provided more context about this decision and how it differs from previous modeling efforts (lines 183-193).**

2.22) L403 – what are “trees” in this context?

***Response: We have added the following sentence to give context for the term “tree” for random forest analysis: “Random forest analysis generates an ensemble of classification or regression trees for a given data set and then combines predictions from the individual trees (Cutler et al. 2007)” (lines 431-432).**

**Works cited in response to reviewers:**

[Lanzas, C., Davies, K., Erwin, S., & Dawson, D. (2020). On modelling environmentally transmitted pathogens. *Interface Focus*, *10*(1), 20190056.](http://paperpile.com/b/Nmqa61/U20j)

[Moorcroft, P. R., & Lewis, M. A. (2006). *Mechanistic Home Range Analysis*. Princeton University Press.](http://paperpile.com/b/Nmqa61/ylIl)
