# Supplementary material for: A mechanistic, stigmergy model of territory formation in solitary animals: Territorial behavior can dampen disease prevalence but increase persistence
Source: PLoS Comput Biol. 2020 Jun 11;16(6):e1007457. doi: 10.1371/journal.pcbi.1007457 (PMC7289346; doi:10.1371/journal.pcbi.1007457)
Supplement: Attachment — Submitted filename: Stigmergy response to reviewers 2020_03_27.docx [file pcbi.1007457.s015.docx]

**March 27, 2020**

**Response to reviewers: PLOS Computational Biology (PCOMPBIOL-D-19-01705R1)**

I thank the authors for their careful attention to the reviewer comments. Based on the comments of R2 I would suggest a few minor changes (detailed below) before recommending this manuscript for publication. With these changes made, I see no reason that this will need to be sent back out to review and I will be happy to direct the manuscript for publication. Specifically:

R2 raises a question about the notation on L216. I assume, from the text, that the sum described in that line is over individuals. However, R2 found that the description left some doubt. Could you please clarify this notation. Perhaps (a suggestion, not a requirement) adding an index j in 1...J to k_x,y to indicate the you are summing the decaying pathogen loads of the J individuals that had previously visited that site?
***Response: Yes, correct—the sum previously described on line 215 is summed over individuals. To address these concerns, we have updated our notation in a couple ways:**

- **Per Reviewer 2’s request, we now refer to** $\boldsymbol{\kappa}_{\boldsymbol{x,y}}\boldsymbol{(t)}$ **as** $\boldsymbol{\kappa(x,t)}$ **and** $\boldsymbol{\eta}_{\boldsymbol{x,y}}\boldsymbol{(t)}$ **as** $\boldsymbol{\eta(x,t)}$ **throughout the text.**
- **On the first mention of pathogen decay, we define** $\boldsymbol{\kappa(x,t)}$ **as the sum of all pathogen loads deposited by *q=1…Q* individuals visiting that particular cell (lines 202-204). We have also updated the notation for time since deposition as being specific to each individual *q* (e.g., *d_q_*). This avoids the confusion of having an index for a summation where the index does not appear in the subsequent expression (Edwards & Auger‐Méthé, 2019), and nicely simplifies our description of the relationship between transmission rate and pathogen load (lines 205-209). We made parallel changes for our description of scent decay rate (lines 151-153).**

Per R1's comment regarding the SIR notation in L203-206, I assume that this mean field notation is presented as a schematic, while actual simulations were run using the site-specific calculations described in the following lines. Could you provide an explicit link between the environmental decay rate \alpha and the decay rate described on L212?
***Response: Yes, we intended that mean field notation would serve as a schematic or overview of the site-specific processes occurring in the simulation. We now clearly delineate that α serves as the exponential rate of pathogen load decay in the ODE model (lines 198-199 & 223-224), and we have updated Table 1 to explicitly state that α and δ are exponential rates of decay. Next, we more clearly link the site-specific description to the ODE models by replacing the state variable *P* with** $\boldsymbol{\kappa}$ **(line 219). Finally, we move this description of the ODE schematic to the end of the transmission section, so that it clearer that this is an overview of these processes rather than an explicit description of site-specific simulations (lines 213-225).**

Change notation on L136 per reviewer's suggestion.
***Response: We have made the suggested edit (line 135).**

You may consider the reviewer's suggestions about citations and make changes if you feel they are helpful, but I will not require any changes at this stage.
***Response: Thank you for the flexibility. Since we added a significant amount of literature in the last revision, we have opted to forgo these final suggestions.**

**Reviewer's Responses to Questions**

Reviewer #1: All my previous comments were excellently addressed. I have no further comments.
***Response: Thank you!**

Reviewer #2: The authors have satisfactorily addressed the majority of my concerns and significantly improved the manuscript. However, some additional problems arise with the added material. Once they are fully addressed in the next revision, the paper could be a valuable contribution to the fields of both movement ecology and disease ecology.
***Response: Thank you! Please see our specific responses to your concerns below.**

Major problem:
1. Notations in the disease model section L203-219 are confusing and not always consistent. Is beta_p a constant as implied? If so, then it appears, based on L203-206, that the loss of susceptible to infection doesn’t depend on pathogen load P. Readers may thus wonder how pathogen load would even affect transmission dynamics. L214 suggests that beta_p is in fact a function of local pathogen load at time t, seemingly notated as k_x,y(t) – it would be more clearly denoted as k(\emph{x},t). However, this dependency is not reflected in the main SIR description, where only the global pathogen dynamics is shown. As a consequence, the equations show no obvious coupling between individual movement and transmission.
***Response: Yes, the transmission rate is a function of pathogen load in the simulations (not a constant). We have updated our notation in the ODE equations by switching out P for** $\boldsymbol{\kappa}$ **to make the link between the ODE approximation and the site-specific simulations clearer (lines 216-219). We have also updated our notation for the transmission rate as** $\boldsymbol{\beta(\kappa)}$ **to reflect that** $\boldsymbol{\beta}$**, is in fact, a function of environmental pathogen load. We hope that this makes the coupling between individual movement and transmission more obvious in the ODE framework. Finally, per reviewer 2’s suggestion, we have updated our notation so that** $\boldsymbol{\kappa}_{\boldsymbol{x,y}}\boldsymbol{(t)}$ **is now** $\boldsymbol{\kappa(x,t)}$ **throughout the text (e.g. line 202).**

L216 is especially confounding. 1) Does P(S->I) denote the probability of a single individual at location \emph{x} and time t going from susceptible to infected, or some changes in global SI proportions as the notation might easily suggest? I assume the former. If so, describing this verbally is highly recommended.

***Response: Yes, we were referring to the probability that a single susceptible individual at location *x* and time** ***t* would progress to being infected. We have made the suggested change by clarifying that *β* serves as the probability of a successful transmission event in a Bernoulli trial (lines 205-207).**

2) What is k(\emph{x},t) being summed over? Individuals? Since this variable measures the existing quantity of pathogen at a site, then it should already include remaining pathogen load from previous visitors, thus making the summation sign unnecessary. Having additional disease modelers read over this section and revise some of the notations may be very useful.

***Response: Thanks for helping us to clarify our mathematical notation. We updated our definition of** $\boldsymbol{\kappa(x,t)}$ **earlier in the text (lines 202-204) to clarify that it reflects the sum of all previous visiting individuals. This also simplifies our description of the probability of transmission nicely to being equal to:** $\boldsymbol{\beta=}\mathbf{min}\boldsymbol{(1, \kappa}\left( \boldsymbol{x,t} \right)\boldsymbol{)}$ **(lines 205-207).**

Minor problem:
L38-41: Two recent paper that are related to this study and may deserve discussion:
- the effects of disease dynamics on movement dynamics: Potts et al. Quantifying behavioral changes in territorial animals caused by sudden population declines. 2013
- the effects of movement dynamics on disease dynamics: Tao et al. Logistical constraints lead to an intermediate optimum in outbreak response vaccination. 2018
***Response: Thank you for your suggestion—these are also nice papers. At this point in the revision, we have 69 references cited in this paper spanning movement and disease ecology, and we believe that we have represented these areas sufficiently.**

L113-116: This material related to population effect still seems beyond the scope of the analysis in this paper. I recommend removing these sentences.
***Response: We have opted to keep the concept of population thresholds in the text because our experimental design does investigate the potential effects of host density on disease dynamics, and indeed, we do find some interesting interactions between stigmergy driven movement, host density, and outbreak characteristics. We already provide more context for the concept of density thresholds is relevant for indirectly transmitted pathogens in the Discussion (lines 350-363).**

L136: notation edit – P(a,b)
***Response: We have made the suggested edit (line 135).**

R2.8 – The model by Bateman et al. (2015) is more appropriately grouped with the other citations in the next sentence instead of with Okubo and Levin (2001).

***Response: We have now moved Bateman et al. (2015) to be grouped with the citations in the next sentence (lines 89-91).**

**References Cited in Response to Reviewers**

Edwards, A. M., & Auger‐Méthé, M. (2019). Some guidance on using mathematical notation in ecology. *Methods in Ecology and Evolution*, *10*(1), 92–99. https://doi.org/10.1111/2041-210X.13105
